# Critical Role of Astrocytic Polyamine and GABA Metabolism in Epileptogenesis

**DOI:** 10.3389/fncel.2021.787319

**Published:** 2022-01-06

**Authors:** Zsolt Kovács, Serguei N. Skatchkov, Rüdiger W. Veh, Zsolt Szabó, Krisztina Németh, Pál T. Szabó, Julianna Kardos, László Héja

**Affiliations:** ^1^Department of Biology, ELTE Eötvös Loránd University, Savaria University Centre, Szombathely, Hungary; ^2^Department of Physiology, Universidad Central Del Caribe, Bayamon, PR, United States; ^3^Department of Biochemistry, Universidad Central Del Caribe, Bayamon, PR, United States; ^4^Institut für Zell- und Neurobiologie, Centrum 2, Charité - Universitätsmedizin Berlin, Berlin, Germany; ^5^Functional Pharmacology Research Group, Institute of Organic Chemistry, Research Centre for Natural Sciences, Eötvös Loránd Research Network, Budapest, Hungary; ^6^MS Metabolomics Research Group, Centre for Structural Study, Research Centre for Natural Sciences, Eötvös Loránd Research Network, Budapest, Hungary

**Keywords:** absence epilepsy, WAG/Rij rat model, APCHA/spermine synthase inhibitor, 4-MCHA/spermidine synthase inhibitor, polyamines in the central nervous system, neurons, astrocytes, glial cells

## Abstract

Accumulating evidence indicate that astrocytes are essential players of the excitatory and inhibitory signaling during normal and epileptiform activity *via* uptake and release of gliotransmitters, ions, and other substances. Polyamines can be regarded as gliotransmitters since they are almost exclusively stored in astrocytes and can be released by various mechanisms. The polyamine putrescine (PUT) is utilized to synthesize GABA, which can also be released from astrocytes and provide tonic inhibition on neurons. The polyamine spermine (SPM), synthesized form PUT through spermidine (SPD), is known to unblock astrocytic Cx43 gap junction channels and therefore facilitate astrocytic synchronization. In addition, SPM released from astrocytes may also modulate neuronal NMDA, AMPA, and kainate receptors. As a consequence, astrocytic polyamines possess the capability to significantly modulate epileptiform activity. In this study, we investigated different steps in polyamine metabolism and coupled GABA release to assess their potential to control seizure generation and maintenance in two different epilepsy models: the low-[Mg^2+^] model of temporal lobe epilepsy *in vitro* and in the WAG/Rij rat model of absence epilepsy *in vivo*. We show that SPM is a gliotransmitter that is released from astrocytes and significantly contributes to network excitation. Importantly, we found that inhibition of SPD synthesis completely prevented seizure generation in WAG/Rij rats. We hypothesize that this antiepileptic effect is attributed to the subsequent enhancement of PUT to GABA conversion in astrocytes, leading to GABA release through GAT-2/3 transporters. This interpretation is supported by the observation that antiepileptic potential of the Food and Drug Administration (FDA)-approved drug levetiracetam can be diminished by specifically blocking astrocytic GAT-2/3 with SNAP-5114, suggesting that levetiracetam exerts its effect by increasing surface expression of GAT-2/3. Our findings conclusively suggest that the major pathway through which astrocytic polyamines contribute to epileptiform activity is the production of GABA. Modulation of astrocytic polyamine levels, therefore, may serve for a more effective antiepileptic drug development in the future.

## Introduction

Polyamines are polycationic molecules that perform various functions in the brain from maintenance of redox balance (Murray-Stewart et al., 2018), through direct regulation of ion channels (Herman et al., 1993; Biedermann et al., 1998; Weiger et al., 1998; Skatchkov et al., 2006; Kucheryavykh et al., 2008; Nichols and Lee, 2018) and various subtypes of glutamate receptors (GluR) (Benveniste and Mayer, 1993; Williams, 1997; Mott et al., 2003), to modulation of higher cognitive functions (Guerra et al., 2016). Polyamines have biphasic effects on GluRs: they either block AMPAR and NMDAR channels at high doses (*reviewed by* Williams, 1997) or activate NMDAR and kainate receptor channels by low doses (Benveniste and Mayer, 1993; Mott et al., 2003). Therefore, direct release of polyamines (as alternative gliotransmitters) from astrocytes may lead to neuronal activity switch (Skatchkov et al., 2014, 2016; Olsen et al., 2015). Indeed, in hippocampal (Ferchmin et al., 1995) and cortical (Rozov and Burnashev, 1999) brain slices, spermine (SPM) produced dramatic changes in the activity of neuronal networks, but understanding of the results was diverse. Polyamines have been suggested to either directly target AMPARs (Rozov and Burnashev, 1999), Ca^2+^ channels (Herman et al., 1993; Ferchmin et al., 1995), NMDARs (Benveniste and Mayer, 1993), Kir channels (Lopatin et al., 1994, 1995; Nichols and Lopatin, 1997; Skatchkov et al., 2000, 2002; Kucheryavykh et al., 2007, 2008), TRPV channels (Ahern et al., 2006), Cx43 GJCs (Skatchkov et al., 2015; Kucheryavykh et al., 2017), or ASIC channels (Duan et al., 2011). Spermidine (SPD) protects from age-related alterations of synapses *via* autophagy mechanism (Sigrist et al., 2014; Maglione et al., 2019), however, data on the release of polyamines from astrocytes with consequent effect on neighboring inhibitory interneurons were reported only in astrocyte culture (Malpica-Nieves et al., 2021).

Importantly, polyamines are predominantly accumulated in astrocytes and other glial cells (Ingoglia et al., 1982; Laube and Veh, 1997; Biedermann et al., 1998; Gilad et al., 1999; Skatchkov et al., 2000). Uptake system for all polyamines, putrescine (PUT), SPD, and SPM, are present in astrocytes (Dot et al., 2000, 2002; Malpica-Nieves et al., 2020, 2021). The accumulated polyamines can also be released from astrocytes through multiple pathways: astrocytic connexin Cx43 hemichannels (Cx43 HCs) (Skatchkov et al., 2010, 2014, 2016; Kirichenko et al., 2021; Malpica-Nieves et al., 2021), vesicular uptake/release mechanisms (Soulet et al., 2004; Hiasa et al., 2014; Takeuchi et al., 2017; Zorec et al., 2018), or by reverse transport *via* polyamine transporters (Makarov et al., 2013).

Putrescine is predominantly converted to SPD and SPM by SPD synthase and SPM synthase, respectively. In addition, polyamines may also be the source of astrocytic GABA synthesis either directly from PUT or when SPD and SPM are catabolized to PUT. Such conversion proceeds *via* a two-step cascade of events: (i) by SPD-SPM acetyl transferase (SSAT) or (ii) by polyamine oxidase and then from PUT *via* monoamine oxidase B (MAOB) and diamine oxidase (DAO) to GABA (Seiler et al., 1973; Testore et al., 1995; Sequerra et al., 2007; Skatchkov et al., 2014; Olsen et al., 2015; Pegg, 2016; Kwak et al., 2020). Since the production of GABA by its common pathway from glutamate *via* glutamic acid decarboxylase is declined by age (Sequerra et al., 2007; Kim et al., 2017), the PUT-derived GABA synthesis is vital in adult (Olsen et al., 2015). Conversion from PUT to GABA was shown in both newborn progenitor cells of subventricular zone (Sequerra et al., 2007) and in adult astrocytes (Kwak et al., 2020).

Previously we revealed (Héja et al., 2009, 2012) that the release of PUT-derived GABA by the reverse operation of astrocytic GABA transporters GAT-2/3 activates extrasynaptic GABA receptors and contributes to tonic inhibition under epileptic conditions. According to this mechanism, the PUT-derived astrocytic GABA provides a negative feedback that combats overexcitation and shortens epileptic seizures (Héja et al., 2012). This Glu/GABA exchange mechanism could be prevented by blocking MAOB and DAO (Héja et al., 2012), suggesting that PUT is a key player in the mechanism. In addition, MAOB and DAO activity may also be decreased by limiting the astrocytic copper concentration as blockade of copper transporter was shown to decrease tonic inhibitory currents (Szabó et al., 2021). Moreover, not only tonic (Héja et al., 2012), but also induced inhibitory synaptic transmission in hippocampal brain slices was depressed by applying alfa-difluoromethylornithine (DFMO), known to block production of PUT (Ferchmin et al., 1995). These data conclusively suggest that PUT significantly modulates seizure generation and maintenance by producing astrocytic GABA, which in turn after released by GAT-2/3 reversal (Héja et al., 2009, 2012; Kirischuk et al., 2012, 2016) or *via* Bestrophin-1 channels (Olsen et al., 2015; Kim et al., 2017; Kwak et al., 2020) significantly contributes to seizure generation and maintenance. It is to note that decreased GABA level in astrocytes produces decreased tonic inhibition not only in epilepsy (but be aware of the altered activity of KCC2 in epilepsy; di Cristo et al., 2018), but also in a mouse model for attention-deficit/hyperactivity disorder (Kim et al., 2017) and Huntington disease (Wójtowicz et al., 2013).

Besides providing GABA, PUT also forms SPD and SPM. Although this process occurs in synapses and some deep brain neurons (Krauss et al., 2006, 2007), the synthesized SPD and SPM are accumulated in glia. In astrocytes, SPM specifically opens astrocytic Cx43 gap junction channels (Cx43 GJCs) (Benedikt et al., 2012) by removal of proton and calcium blocks in Cx43 channels (Skatchkov et al., 2015; Kucheryavykh et al., 2017). These channels, formed from two coupled Cx43 HCs (Contreras et al., 2003), enable physical coupling between adjacent astrocytes and allow synchronization of the astrocytic syncytium. Also, SPM passes through the open Cx43 GJCs (Benedikt et al., 2012), thereby including additional astrocytes in the iso-potential network (Ma et al., 2016) due to SPM-dependent coupling. This observation points to a crucial role for SPM in astrocytic syncytium formation. Cx43 GJCs play a pro-epileptic role in the *in vitro* low-[Mg^2+^] temporal lobe epilepsy (TLE) model, but intriguingly they play an antiepileptic role in the *in vivo* absence epilepsy model WAG/Rij rats (Vincze et al., 2019). It is therefore plausible to hypothesize that the astrocytic syncytium synchronized by SPM signaling through Cx43 GJCs may contribute to the genesis of epileptic activity (Russo et al., 2016).

Using several approaches in this work, we asked how polyamines may affect neuron-glia coupling, interact with GABA transmissions, and modulate neuronal excitation and synaptic activity under epileptic conditions. We used different agents to (i) inhibit the synthesis of SPM and SPD by 3-(aminopropyl)cyclohexylamine (APCHA) and trans-4-methylcyclohexylamine (4-MCHA), respectively; (ii) activate Cx43 GJC coupling and probably GluRs by exogenous SPM; (iii) stimulate the release of polyamines by specific depolarization of astrocytes with the specific gliotoxin mono-fluoroacetate (FA); (iv) increase the surface expression of GAT-2/3 by levetiracetam in the presence and absence of GAT-2/3 blocker [(S)-1-[2-[tris(4-methoxyphenyl)methoxy]ethyl]-3-piperidinecarboxylic acid (SNAP-5114).

## Materials and Methods

### Animals

Animals were kept and used in accordance with standard ethical guidelines and approved by the local Animal Care Committee, the Government Office for Pest County (reference numbers PEI/001/3671-4/2015 and PE/EA/3840-4/2016), the Hungarian Act of Animal Care and Experimentation (1998, XXVIII, section 243), European Communities Council Directive 24 November 1986 (86/609/EEC), and EU Directive 2010/63/EU on the use and treatment of animals in experimental laboratories. The experiments on WAG/Rij rats were approved by the Animal Care and Experimentation Committee of the Eötvös Loránd University (Savaria University Centre) and National Scientific Ethical Committee on Animal Experimentation (Hungary) under license number VA/ÉBNTF02/85-8/2016. All efforts were made to reduce animal suffering and the number of animals used. The experiments on Sprague-Dawley rats were carried out in accordance with a protocol approved by the Universidad Central del Caribe Institutional Animal Care and Use Committee (UCC, Bayamon, PR, United States) (protocol numbers: #018-2021-05-010 and #018-2021-04-00, approval date: March 2021). All animals were housed in groups of 3–4 under standard laboratory conditions (free access to water and food; 12:12 h light-dark cycle, light was on from 08.00 a.m. to 08.00 p.m.; air-conditioned room at 22 ± 2°C). In total, 6 Sprague-Dawley rats were used to determine the effect of SPM on network activity, 23 Wistar rats were used for the *in vitro* epilepsy measurements and 48 WAG/Rij rats were used for the *in vivo* epilepsy measurements.

### Solutions

#### Artificial Cerebrospinal Fluid

Artificial cerebrospinal fluid (ACSF) contained in mM: 129 NaCl; 3 KCl; 1.6 CaCl_2_; 1.8 MgSO_4_; 1.25 NaH_2_PO_4_; 21 NaHCO_3_; 10 glucose. To induce epilepsy, MgSO_4_ was eliminated and 2 mM KCl was added (low-[Mg^2+^] ACSF). In the *in vitro* experiments, SPM (SPM 200 μM) and FA sodium salt (1 mM) were diluted in ACSF or low-[Mg^2+^] ACSF. The pH value of 7.4 was not affected by the applied concentrations. All solutions were continuously oxygenated (95% O_2_, 5% CO_2_). In the *in vivo* experiments on WAG/Rij rats, levetiracetam (intraperitoneal/i.p. 200 mg/kg; TCI, Tokyo, Japan), DFMO (i.p. 150 mg/kg; TCI, Tokyo, Japan), 4-MCHA (i.p. 25 mg/kg), and APCHA (i.p. 25 mg/kg; TCI, Tokyo, Japan) were dissolved in saline. It was demonstrated previously that 1–30% dimethyl sulfoxide (DMSO) solution did not change absence epileptic activity in WAG/Rij rats (Kovács et al., 2011), thus, SNAP-5114 (i.p. 20 mg/kg; TOCRIS, Bristol, United Kingdom) was dissolved in 10% DMSO solution. Unless otherwise stated, all drugs were purchased from Sigma-Aldrich, Budapest, Hungary, and Saint Louis, MO, United States.

### Slice Preparation

#### Rat Brain Slices for Low-[Mg^2+^] Epilepsy Model Measurements

Transverse, 400 μm thick, hippocampal-entorhinal slices from 10 to 15 day old Wistar rats (Toxicoop, Budapest, Hungary) were prepared in modified ACSF (75 mM sucrose; 87 mM NaCl; 2.5 mM KCl; 0.5 mM CaCl_2_; 7 mM MgSO_4_; 1.25 mM NaH_2_PO_4_; 25 mM NaHCO_3_; 25 mM glucose) at 4°C. The slices were incubated in an interface-type chamber in continuously oxygenated ACSF for 1 h at 37°C followed by incubation in room temperature before performing the experiments.

#### Rat Brain Slices for Extracellular Paired-Pulse Facilitation Recordings

Female Sprague-Dawley rats of about 145 g were used. The rats were bred and sacrificed following procedures approved by the Institutional Animal Care and Use Committee. After decapitation, the brains were removed and the hippocampi dissected on ice while irrigated with ice-cold ACSF. The composition of the ACSF was (in mM): NaCl 125, KCI 2.5 (and 5 mM KCl for PPF induction), NaH_2_PO_4_ 1.25, MgSO_4_ 2, CaC1_2_ 1.8, and glucose 10. Transverse slices (400 μM) were obtained using a manual slicer with micrometer scale adjustment. The slices were promptly transferred to an interface recording chamber where they were incubated 1 h before recording on a nylon support at the interface of humidified 95% O_2_, 5% CO_2_, and ACSF. The temperature of the chamber was kept at 34.5°C.

#### Sprague-Dawley Rat Brain Slices for Patch Clamp Recordings

Transverse, 300 μm thick hippocampal slices were prepared from the brains of Sprague-Dawley rats of both sexes (age P20-P30) and were dissected in ice-cold ACSF saturated with 95% O_2_, 5% CO_2_. The slices were cut using a vibratome (VT1000S; Leica, Nussloch, Germany) and incubated for recovery in a standard ACSF solution containing (mM) 127 NaCl, 2.5 KCl, 1 MgCl_2_, 2 CaCl_2_, 1.25 NaH_2_PO_4_, 10 glucose, 26 NaHCO_3_, gassed with 5% CO_2_/95% O_2_, pH 7.4, at 35°C for 20 min, and then at room temperature (osmolarity: 305 mOsm/l). After 30 min of total incubation, the slices were placed in a recording flow chamber (0.5 mL volume) and superfused continuously with oxygenated ACSF at room temperature (23–24°C, 1 mL/min). Whole cell recording and fluorescent dye tracing studies were performed as described previously (Benedikt et al., 2012).

### Immunocytochemistry in Brain Slices

Subsequent to extracellular paired-pulse facilitation recordings, the slices were fixed by immersion into a solution of 4% paraformaldehyde, 0.05% glutaraldehyde, and 0.2% picric acid in 0.1 M phosphate buffer, pH 7.4 (Somogyi and Takagi, 1982). Fixation was followed by 0.15 M sucrose in 0.1 M phosphate buffer, pH 7.4. The slices were then shock-frozen after 48 h pretreatment with 0.8 M sucrose and stored for up to 12 months at −80°C. Immunoenzymatic staining was performed as described previously for brain (Laube and Veh, 1997) and retina (Biedermann et al., 1998; Skatchkov et al., 2000). In short, from fixed and frozen brain 400 μm slices, 25 μm sections were obtained using a cryostat. The sections were pretreated with 1% sodium borohydride in PBS for 15 min and subsequently permeabilized with 0.3% Triton X-100 for 30 min. After incubation with primary antibody (affinity purified anti-SPM antibody) for 24 h at room temperature, free floating sections were treated with secondary antibody (biotinylated goat anti-rabbit IgG, 1:2,000, Vector/Camon, Wiesbaden) for 18 h and with an ABC complex (Vectastain Elite, 1:1,000, Vector/Camon, Wiesbaden) for 6 h. Peroxidase activity was revealed with 1.4 mM 3,3’-diaminobenzidine, 10 mM imidazole, 0.3% nickelous ammonium sulfate, and 0.015% H_2_O_2_ in 50 mM Tris/HCl, pH 7.6 for 3 min at room temperature. For immunization, a bovine serum albumin (BSA) hapten conjugate (Meyer et al., 1991) was obtained by coupling SPM to BSA using glutaraldehyde. The anti-SPM antibody was raised in rabbits, affinity purified, and characterized as described previously (Laube and Veh, 1997). It recognizes glutaraldehyde-linked/fixed SPM and SPD with similar efficiency, cross reacts weakly with fixed put, and shows negligible activity against lysine, arginine, ornithine, histamine, and ethanolamine (Laube and Veh, 1997). In controls, the sections were incubated either without primary antibody or antisera were blocked by preincubation with the SPM-BSA conjugate (10 μg/ml), 2 h prior to addition to sections. In some sections cell bodies were counterstained with 0.8% methyl green in 20% aqueous ethanol for 30 min at room temperature. This procedure was also used in retina of different species including human retinas (Biedermann et al., 1998; Skatchkov et al., 2000) and in brain slices (Skatchkov et al., 2014, 2016).

### Electrophyisology

#### *In vitro* Population Spike Recording

**FIGURE 1:**
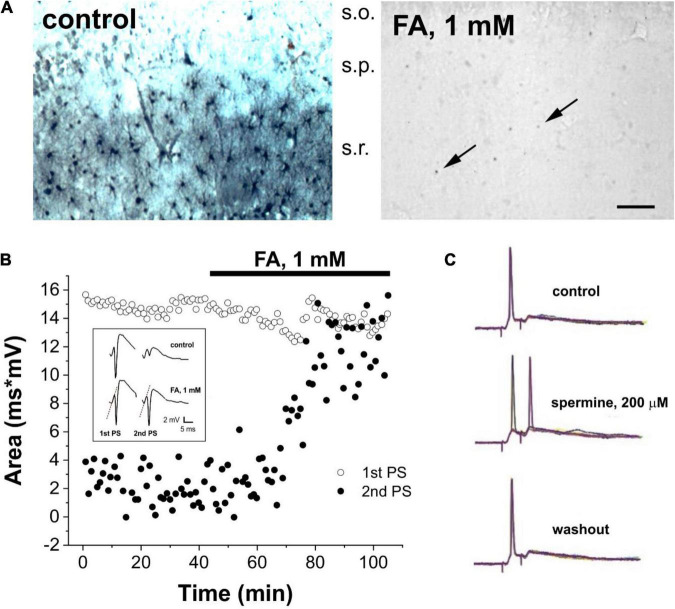
Glial status determines neuronal behavior in Sprague-Dawley rat brain slices. **(A)** Immunocytochemical localization of spermine (SPM) in the CA1 region of the hippocampus under control conditions (*left*) and following 40 min perfusion with 1 mM of the gliotoxin monofluoroacetate (FA) (*right*). Note that in the left image, the pyramidal cell layer (s.p.) is rather empty, the *stratum radiatum* (s.r.) displays numerous SPM-positive astrocytes. In the right image, after perfusion with gliotoxin FA, SPM-positive astrocytes have completely disappeared in the *stratum radiatum*, leaving back only a few weakly positive cell bodies (arrows). S.o.: *stratum oriens*. Bar indicates 30 μm for both images. **(B)** Time course of the area of first (open circles) and second (black circles) population spikes (PSs) recorded from CA1 pyramidal cell layer in response to paired-pulse stimulation (20 ms inter-pulse intervals each minute) in the presence of 1 mM FA. Individual field potential recordings are shown in the inset. **(C)** Average of 10 whole cell patch clamp recordings from CA1 pyramidal cells in response to paired-pulse stimulation (15 ms inter-pulse intervals each minute) under control conditions (*top*), in the presence of 200 μM spermine (*middle*) and during washout (*bottom*).

We used modified method from Teyler (1980) and Ferchmin et al. (1993, 1995). Briefly, brain 400 μm slices after 1 h incubation were used for the stimulation that was delivered with concentric bipolar electrodes. Constant current stimuli were generated by a S48 stimulator and PSIU6 stimulus isolation unit (Grass, United States). The recording electrodes were filled with 2 M NaCI glass micropipettes with impedance ranging from 3 to 5 MOhm. Paired stimuli of 0.2 ms duration and 20 ms interpulse interval were delivered in *stratum (s.) radiatum* Schaeffer collaterals every minute. The average strength of the stimuli was set to obtain 50–60% of the maximal response, values ranged from 50 to 170 μA. The recording electrodes were placed in *s. radiatum* and in *s. pyramidale*. The PS responses were amplified (P511 Grass amplifier), digitized, and stored for further analysis with the LABMAN system. The area (ms × mV) of the PS, recorded in *s. pyramidale*, and the initial slope of the field EPSP (mV/ms) from *s. radiatum* are the variables presented (Figures 1B,C).

#### *In vitro* Patch Clamp Recording From Pyramidal Neurons

Two micromanipulators [MX7500 with MC-1000 drive (Siskiyou Inc., Grants Pass, Oregon, United States)] were used for whole cell voltage-clamp and current-clamp recording and for positioning micropipettes. Pyramidal neurons from CA1 area were clamped using patch pipettes made from borosilicate glass tubing OD 1.5 mm, ID 1.0 mm (World Precision Instruments, Sarasota, Florida, United States) pulled in four steps using a Faming-Brown P-97 pipette puller (Sutter Instruments Corporation, Novato, California, United States) and filled with intracellular solution (ICS) containing in mM: 117 K-gluconate, 13 KCl, 2 MgCl_2_, 10 HEPES, pH adjusted to 7.2 with KOH (osmolarity 275 mOsm/l). After filling with ICS, the final micropipette resistance was close to 6 MΩ, which was optimized for recordings to achieve seals of more than 3 GΩ on cell membranes. Cells were visualized and identified using several procedures: infrared, black and white, and color confocal microscopy. We used an Olympus infrared microscope (BX51WI; Olympus, Shinjuku-ku, Tokyo, Japan) equipped with a 40X water immersion objective and two cameras: for infrared differential interference contrast (IR-DIC) with DIC optics and camera (IC-73) and for fluorescent images with a second camera (DP30BW digital, Olympus, Shinjuku-ku, Tokyo, Japan). DP controller software (Olympus) was used to visualize and to record black and white images. Morphologically and electrophysiologically distinguished pyramidal cells were used. We used Multiclamp 700A patch clamp amplifier with a DigiData 1440A interface (Molecular Devices Inc., Sunnyvale, California, United States). The pClamp 10 software package (Molecular Devices Inc.) was used for data acquisition and analysis. These patch clamp recordings are presented in Figure 1C.

#### Low-[Mg^2+^] Epilepsy Model

The electrophysiological field potential (FP) recordings were performed at 31°C. For FP recordings, glass microelectrodes (1–4 MΩ) were filled with ACSF solution and were inserted in the CA3 *stratum pyramidale*. The signals were recorded with Multiclamp 700A amplifiers (Axon Instruments, Foster City, CA, United States), low-pass filtered at 2 kHz and digitized at 10 or 20 kHz (Digidata 1320A, Axon Instruments). The recordings were analyzed after high pass filtering at 1 or 2 Hz. Epileptiform activity was induced by switching the perfusing solution (ACSF) to low-[Mg^2+^] ACSF (ACSF with no added MgSO_4_ and KCl elevated to 5 mM). To test the effect of the drugs on the appearance of seizure-like events (SLEs), they were continuously present in the ACSF and low-[Mg^2+^] ACSF solutions. First, normal ACSF solution was applied for 20 min as control condition. Then normal ACSF solution with 4-MCHA, APCHA, or SPM was applied for further 20 min. Finally, low-[Mg^2+^] ACSF, containing the same concentration of drugs was applied.

#### Electroencephalogram Recording in the WAG/Rij Rat Model of Absence Epilepsy

Female WAG/Rij rats (8–9 months old, 179–198 g; breeding colony of WAG/Rij rats at ELTE Savaria University Centre, Szombathely, Hungary) were implanted in isoflurane-air mixture (2.0–2.5%) anesthesia for *in vivo* experiments. Screw electrodes were implanted into the bone above frontal cortex (AP: 2.0 mm and L: 2.1 mm) and parietal cortex (AP: -6.5 mm and L: 2.1 mm) (Paxinos and Watson, 2007) for EEG recording. A ground electrode was placed above the cerebellar cortex, whereas the one side insulated reference electrode (a 3 × 4 mm stainless steel plate) was implanted under the skin and over the masseter muscle. The plate and electrodes were soldered to a 10-pin socket and fixed to the skull by dentacrylate cement (Ivoclar, Liechtenstein). Lidocaine ointment (5%; EGIS, Hungary) was used for postoperative pain relief (Kovács et al., 2006). After implantations, all the rats were allowed to recover for 2 weeks.

Electroencephalogram (EEG) was recorded by a differential biological amplifier (Bioamp4, Supertech Ltd., Pécs, Hungary), which was attached to a data capture and analysis device (CED 1401 mkII, Cambridge Electronic Design Ltd., United Kingdom). The bandwidth of the EEG recording was 0.16–150 Hz and it was sampled at 1 kHz sampling rate (Kovács et al., 2014). Handling may evoke stress-induced changes in behavior for about 30 min, which can modify SWD number (Coenen and van Luijtelaar, 2003; Kovács et al., 2015). Thus, the evaluation of SWD parameters was carried out between 30 and 210 min of recording period between 3:00 p.m. and 6:00 p.m. Normal grooming and behavior were observed in all animals 20–25 min after the drug administration and the connection of rats to the biological amplifier.

To adapt the WAG/Rij rats to the experimental procedures, all the animals were handled daily and were connected to the biological amplifier for 3 days (adaptation period) after 2 weeks recovery period. After the adaptation period, the rats were assigned into six groups. To establish averaged control SWD number and SWD time, the rats were i.p. injected once per day by saline (1 ml/kg body weight; first, second and sixth group) or twice per day by saline (1 ml/kg body weight/1st injection and, 30 min later, it was followed by a same saline injection/2nd injection; third, fourth and fifth group) on 3-day control period. After 3-day control periods, the first group of animals (*n* = 8) received i.p. APCHA (25 mg/kg in 1 ml/kg saline) on the fourth day, whereas a mixture of APCHA (25 mg/kg) and 4-MCHA (25 mg/kg) in 1 ml/kg saline was i.p. injected on the fifth day. In relation to second group (*n* = 8), the treatment of animals was similar to first group on the fifth day but the i.p. injection contained 4-MCHA (25 mg/kg in 1 ml/kg saline) on the fourth day. After control periods, the animals in third and fourth group received two i.p. injections (1st injections were followed by 2nd injections 30 min later) for 5 consecutive days. In relation to third group (*n* = 12), i.p. saline (1 ml/kg, 1st injection) and levetiracetam (200 mg/kg in 1 ml/kg saline; 2nd injection) were injected. In the fourth group (*n* = 12), similar treatment to third group was carried out between 1st and 4th treatment days, but a combined injection of SNAP-5114 (i.p. 20 mg/kg in 1 ml/kg 10% DMSO solution; 1st injection) with levetiracetam (200 mg/kg in 1 ml/kg saline; 2nd injection) were used on the 5th treatment day. To investigate the effect of SNAP-5114 alone on SWD number, after 3-day control periods, the fifth group of animals (*n* = 8) received SNAP-5114 (i.p. 20 mg/kg in 1 ml/kg 10% DMSO solution; 1st injection) and, 30 min later, 1 ml/kg saline was i.p. injected (2nd injection) on the fourth day. After control period, the animals in group 6 (*n* = 6) were i.p. injected by DFMO (150 mg/kg in 1 ml/kg saline) on the fourth day. EEGs were recorded every day.

### Mass Spectrometry

After preincubation for 1 h in an interface-type incubation chamber, five 300 μm hippocampal-entorhinal slices from 10 to 15 day old Wistar rats were placed on the bottom of a well in a 24-well plate. Following 1 h incubation in 300 μl of either normal ACSF or low-[Mg^2+^] ACSF in the absence or presence of 250 μM 4-MCHA, the bath solution was removed and used as a measure of the extracellular solution. The slices were transferred to an eppendorf tube. The remaining small amount of buffer was removed from the tube and the slices were weighted to obtain the wet tissue weight.

A QTRAP 6500 triple quadrupole, linear ion trap mass spectrometer equipped with a Turbo V Source (Sciex, MA, United States), and an Agilent 1100 Series HPLC (Agilent, CA, United States) were used for LC-MS/MS analysis. Chromatographic separation was carried out on an Agilent Zorbax Rx-SIL column (250 × 4.6 mm, 5 μm, Kromat Ltd., Hungary). Water containing formic acid in 0.1 V/V% and acetonitrile containing formic acid in 0.1 V/V% was used in inverse gradient mode for separation. The flow rate was 1 ml/min and 5 μl of the samples were injected. The column temperature was ambient, and the samples were kept at 10°C in the autosampler during the acquisition. Electrospray ionization was performed in positive mode. The MS/MS was operated under multiple reaction monitoring (MRM) mode with nitrogen as collision gas. The MRM quantifier transitions (Q1/Q3) for the components are: GABA: 104.0/86.9, SPD: 146.1/71.8, SPM: 203.1/111.9, and PUT: 89.0/29.9.

### Data Digitization and Processing

The Clampfit (Axon Instruments) program was used to evaluate electrophysiological data. The recordings were analyzed after high pass filtering at 1 or 2 Hz. SLE onset was determined by the negative FP deflection and a high-frequency oscillation at the start of discharges. This is the paroxysmal initiation period, which is followed by the tonic and clonic periods of paroxysmal spike discharges (Lasztóczi et al., 2004). The interictal period was determined as the time from the end of a given SLE to the beginning of the next SLE. Being not fully developed, the first SLE in each slice (SLE0) was discarded from data evaluation. Data are shown as mean ± SEM and were analyzed with one-way ANOVA (OriginPro, 2018). Statistical significance was considered at *p* < 0.05.

SWDs can be characterized by 7–11 Hz discharge frequency within SWDs, 1–50 s duration and 0.2–1.0 mV amplitude (Coenen and van Luijtelaar, 2003). Moreover, the SWDs contain a train of asymmetric spikes and slow waves starting and ending with sharp spikes and the average amplitude of SWDs is at least twice as high as the basal EEG activity. EEG recordings were split into 30 min sections and the features of SWDs above were used for automated separation of SWDs in the EEG files (all of automatically selected SWDs were confirmed by manual supervision). All results are expressed as means ± SEM and data analysis was performed similar to the *in vitro* results.

### Statistics

All statistical analyses were performed using Matlab, Origin 2021, Sigma Plot, and GraphPad Prism [version 8.4.3 (471), San Diego, CA, United States]. Data are reported as mean ± SEM. Significant differences between groups were evaluated using Student’s paired *t-*test or two-way ANOVA. Statistical significance was accepted for *P* < 0.05.

## Results

### Exogenous Spermine Facilitates Neuronal Activity *in vitro* Under Physiological Conditions

Since extracellular SPM significantly changes neurotransmission in brain slices by converting paired-pulses inhibition (PPI) to facilitation (PPF) (Ferchmin et al., 1995; Rozov and Burnashev, 1999; Shin et al., 2005; Skatchkov et al., 2014, 2016; Hülsmann et al., 2017) and the metabolic status of astrocytes produces strong changes in synaptic neuronal activity (Keyser and Pellmar, 1994, 1997; Fonnum et al., 1997), we assessed whether these mechanisms are mediated by polyamines. In this study, we show that SPM is exclusively localized to astrocytes in the CA1 region of the hippocampus (Figure 1A left panel). Perfusion with the gliotoxin FA triggered the release of astrocytic SPM (Figure 1A right panel). To evaluate the effect of the released SPM, we measured PPI and PPF. When Schaffer collaterals were stimulated by 10–25 ms interval in normal ACSF containing 2.5 mM K^+^, pyramidal cells showed normal first response (PS) and depressed second one due to the activation of inhibitory GABAergic interneurons in stratum radiatum, which block pyramidal cells. Such network is developed after six postnatal days (Harris and Teyler, 1983) and is sensitive to polyamine synthesis, particularly when PUT synthesis was blocked by DFMO (Ferchmin et al., 1993). Application of the specific gliotoxin FA (1 mM) (Keyser and Pellmar, 1994, 1997; Fonnum et al., 1997) resulted in disinhibition by converting PPI to PPF (Figure 1B), most probably due to SPM release (Figure 1A). Indeed, direct application of 200 μM SPM (Figure 1C) also had a strong disinhibitory effect, transforming PPI into PPF. Note that the first PS(1st PS) and population excitatory synaptic potentials were not affected by FA application (Figure 1B, dotted lines in inset), demonstrating that it does not directly affect excitatory inputs on pyramidal cells. Therefore, these data suggest that depolarized glial cells rapidly release a modulator to neighboring inhibitory interneurons that regulate inhibitory feedback to pyramidal cells.

### Both Spermine Application and Spermine Synthesis Inhibition Attenuate Epileptiform Activity in the Low-Mg^2+^ Temporal Lobe Epilepsy Model *in vitro*

Astrocytic concentrations of polyamines may impact epileptic activity in various ways. PUT acts as a precursor for astrocytic GABA synthesis. Since astrocytic GABA can be released through GABA transporters GAT-2 and GAT-3 during epileptiform activity (Héja et al., 2009, 2012), PUT catabolism to GABA is expected to be anticonvulsive. In contrast, conversion of PUT to SPD and SPM is expected to be pro-convulsive due to the dis-inhibitory effect of SPM (Figure 1) and its ability to keep astrocytic Cx43 GJCs open (Skatchkov et al., 2015), which may facilitate epileptogenesis in the low-[Mg^2+^] TLE model *in vitro* (Kékesi et al., 2015; Vincze et al., 2019).

**FIGURE 2:**
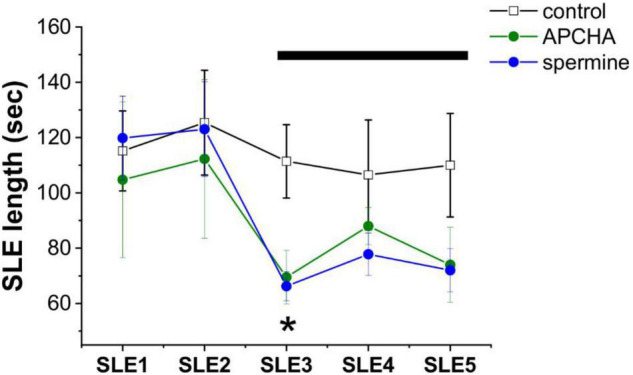
Inhibition of spermine synthesis and exogenous spermine addition both inhibits seizure-like events in the *in vitro* low-[Mg^2+^] model of temporal lobe epilepsy. Length of seizure-like events (SLEs), measured in the CA3 pyramidal layer of hippocampal slices under control conditions and in the presence of the spermine synthase inhibitor 3-(aminopropyl)cyclohexylamine (APCHA) (200 μM) or exogenous spermine (200 μM) (*n* = 4–6 animals). Drugs were applied after two fully developed SLEs in the low-[Mg^2+^] ACSF. Bar shows the application period of APCHA or spermine. Asterisks denote significant (*p* < 0.05) difference from the control.

To assess the contribution of these routes to epileptogenesis, we measured the effect of exogenously applied SPM as well as inhibition of SPM synthesis on the appearance of SLEs in the low-[Mg^2+^] TLE model *in vitro*. We observed that inhibition of SPM synthesis by APCHA (250 μM) significantly (*p* = 0.04) reduced SLE length (Figure 2), possibly due to the resulting enhancement of PUT to GABA conversion and consequent GABA release (Héja et al., 2012). It is also plausible that the decreasing SPM concentration leads to reduced opening of astrocytic gap junctions (Kucheryavykh et al., 2017), which diminishes astrocytic and neuronal synchronization (Vincze et al., 2019). Interestingly, however, application of exogenous SPM also resulted in decreased SLE activity (Figure 2) (*p* = 0.02), despite the excitatory nature of direct SPM application under physiological conditions (Figure 1C). This observation suggests that the major route by which polyamines modulate network activity under epileptic conditions is the enhancement of astrocytic GABA concentration that can be achieved both by inhibiting SPM synthesis and by enhancing the SPM to PUT to GABA catabolic pathway in response to increased SPM concentration.

### Effect of Polyamine Metabolism on Epileptiform Activity in the WAG/Rij Rat Model of Absence Epilepsy

To assess the impact of polyamine metabolism on epileptic activity *in vivo*, we investigated SWDs in WAG/Rij rats under control conditions and in the presence of SPD synthase or SPM synthase inhibitors. WAG/Rij rats were chosen because they are well-studied (Coenen and van Luijtelaar, 2003; Russo et al., 2016), extensively used for investigation of not only pathophysiology of absence epilepsy but also effects of drugs and therapeutic tools on different central nervous system (CNS) diseases, and they generate seizures spontaneously, thus drug treatment is not needed to trigger epileptic seizures (Coenen and van Luijtelaar, 2003; Russo and Citraro, 2018). Since SPD forms both by *de novo* synthesis from PUT and by catabolic degradation of SPM to SPD (Seiler, 1990), we also applied the SPD and SPM synthase inhibitors in combination to effectively reduce the concentration of both polyamines.

**FIGURE 3:**
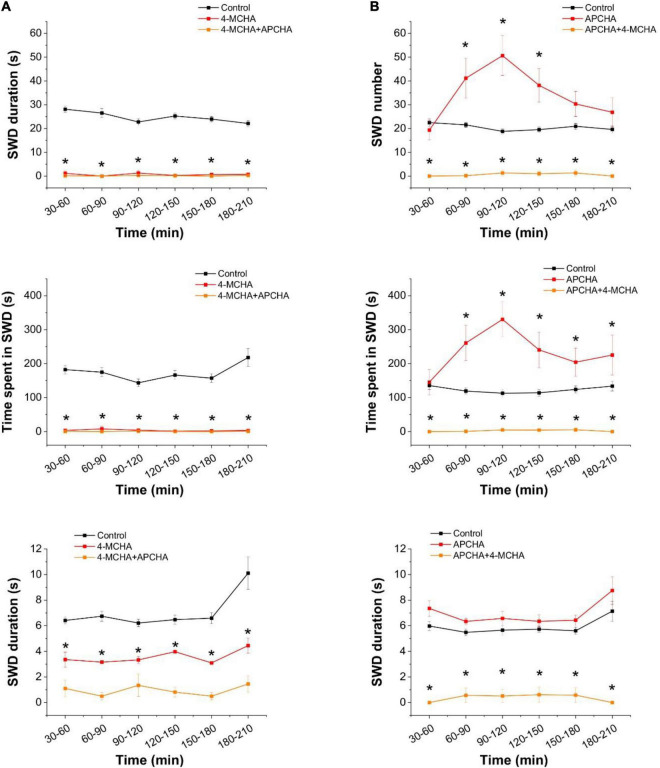
Inhibition of spermidine synthesis blocks, inhibition of spermine synthesis enhances the appearance of spike-wave discharges in the *in vivo* WAG/Rij rat model of absence epilepsy. **(A)** Effect of the spermidine synthase inhibitor 4-MCHA (25 mg/kg) on SWD number (top), total SWD time (center), and average SWD duration (bottom). **(B)** Effect of the spermine synthase inhibitor APCHA (25 mg/kg) on SWD number (top), total SWD time (center), and average SWD duration (bottom) (*n* = 8 animals in each group). Asterisks denote significant (*p* < 0.05) difference from the control.

We observed that 4-MCHA, a selective SPD synthase inhibitor (Shirahata et al., 1993), almost completely eliminated SWD activity and significantly reduced the duration of the remaining few seizures (Figure 3A). In contrast, the selective SPM synthase inhibitor APCHA (Shirahata et al., 1993) temporarily increased SWD activity without affecting their durations (Figure 3B). Since the combined application of 4-MCHA and APCHA also resulted in the blockade of SWDs, the transient pro-epileptic effect of APCHA may be attributed to the increased SPD level that compensates the decrease in SPM concentration. In addition, inhibition of SPM synthase may also lead to a reduction in SPM-mediated opening of astrocytic Cx43 GJCs, which is known to contribute to epileptic activity in WAG/Rij rats (Vincze et al., 2019).

**FIGURE 4:**
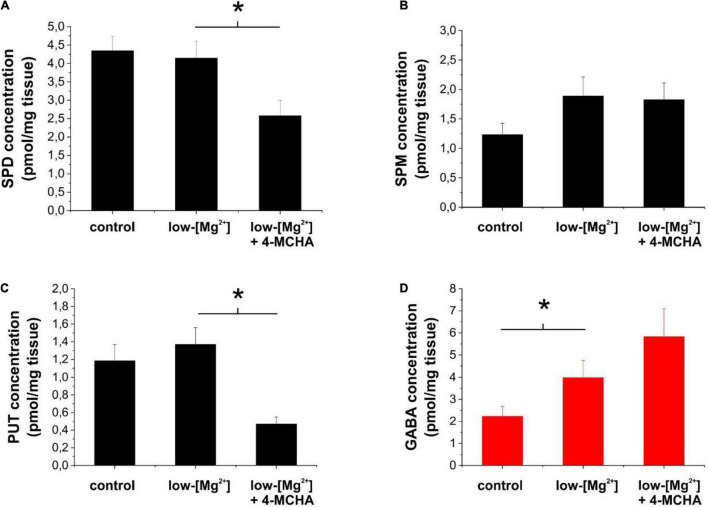
Inhibition of spermidine synthesis decreases extracellular polyamine concentrations, but increases extracellular GABA concentration in brain slices. **(A–D)** Concentrations of spermidine **(A)**, spermine **(B)**, putrescine **(C)**, and GABA **(D)** in the bathing medium of hippocampal slices following 1 h incubation in normal ACSF (control) or in low-[Mg^2+^] ACSF in the absence and presence of 250 μM 4-MCHA as determined by LC-MS (*n* = 8 animals). Asterisks denote significant difference (*p* < 0.05).

To interpret the above results, we measured the changes in the extracellular concentrations of polyamines and GABA in response to inhibition of SPD synthesis in brain slices during SLEs. Previously, we demonstrated that under this condition, GABA is released from astrocytes through GAT-2/3 transporters (Héja et al., 2012). In this study, we applied 250 μM 4-MCHA and determined the extracellular level of PUT, SPD, SPM, and GABA by mass spectrometry. We observed that 4-MCHA significantly decreased the extracellular concentration of SPD (Figure 4A, *p* = 0.007), while extracellular SPM concentration was not affected (Figure 4B). Interestingly, PUT concentration also significantly decreased following SPD synthase blockade (Figure 4C, *p* < 0.001), despite the reduced PUT to SPD conversion. This observation can be explained by the enhanced conversion of PUT to GABA, as indicated by the increased extracellular GABA level (Figure 4D). In summary, 4-MCHA application leads to increased GABA concentration and consequently enhanced tonic inhibition, which may explain its antiepileptic effect (Figure 3A).

### GAT-2/3-Mediated Astrocytic GABA Release Significantly Contributes to the Emergence of Seizures in the WAG/Rij Rat Model of Absence Epilepsy

**FIGURE 5:**
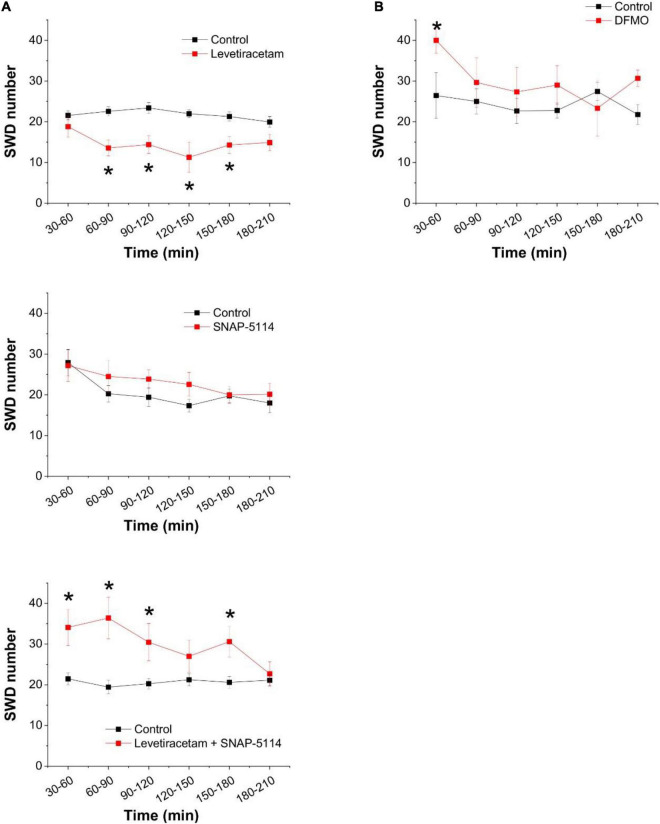
Increased astrocytic GABA release through GAT-2/3 transporters suppresses spike-wave discharges in the *in vivo* absence epilepsy model WAG/Rij rat. **(A)** Effect of the levetiracetam treatment (i.p. 200 mg/kg/day, 5 consecutive days, *top*, *n* = 12 animals) and the specific GAT-2/3 inhibitor SNAP-5114 (i.p. 20 mg/kg, *center*, *n* = 8 animals) on SWD numbers. Effect of SNAP-5114 (i.p. 20 mg/kg) applied on 5th day of levetiracetam treatment (i.p. 200 mg/kg, *bottom*, n = 12 animals) on SWD number. **(B)** Effect of the ornithine decarboxylase inhibitor alpha-difluoromethylornithine (DFMO, 150 mg/kg) on SWD number (*n* = 6 animals). Asterisks denote significant difference from control (*p* < 0.05).

Since the inhibition of SPD synthase inevitably leads to accumulation of PUT, and this PUT may contribute to GABA formation and its release through GAT-2/3 transporters, we evaluated the effect of the FDA-approved antiepileptic drug levetiracetam that is known to increase the surface expression of GAT-2/3. Treatment of WAG/Rij rats with levetiracetam through a course of 5 days significantly suppressed epileptic activity (Figure 5A top panel). To confirm that the observed antiepileptic effect of levetiracetam is due to its ability to increase GAT-2/3 expression, in a separate experiment, we blocked GAT-2/3 by their specific inhibitor SNAP-5114. Application of SNAP-5114 only slightly increased SWD appearance when applied alone (Figure 5A center panel). However, in levetiracetam-treated animals, SNAP-5114 completely reversed the antiepileptic effect of levetiracetam, leading to significantly increased appearance of seizures (Figure 5A bottom panel), indicating that levetiracetam largely increased GAT-2/3 expression and overexpression of GAT-2/3 has been anticonvulsive.

After concluding that the PUT to GABA conversion plays a prominent role in the absence epilepsy as well, we also investigated whether direct modulation of PUT concentration is an effective way to adjust seizure activity. To this end, we blocked ornithine decarboxylase activity by DFMO. Interestingly, however, direct inhibition of PUT synthesis did not affect SWDs significantly (Figure 5B). These findings suggest that PUT is not synthesized from ornithine in these adult animals, instead, it was made available from external sources.

## Discussion

**FIGURE 6:**
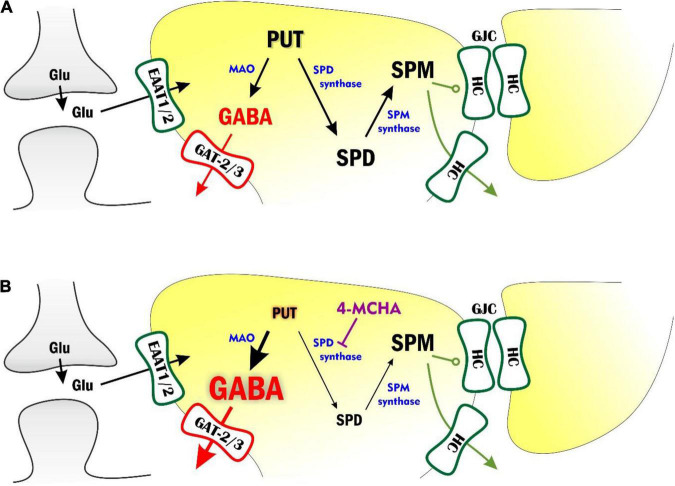
Modulation of polyamine metabolism and astrocytic GABA pathway can attenuate epileptiform activity through multiple pathways. **(A)** Astrocytic polyamine metabolism affects network excitation by multitude of ways. Putrescine (PUT) can be converted to GABA by monoamino oxidases (MAO). The astrocytic GABA is subsequently released by the reverse operation of GAT-2/3 transporters and increases tonic inhibition on neurons. PUT can also be metabolized to spermidine (SPD) and spermine (SPM). SPM, on the other hand, keeps open astrocytic gap junctions, formed from connexin hemichannels (HCs) and can also release and facilitate neuronal activity. **(B)** Inhibition of SPD synthase would increase astrocytic PUT concentration. However, according to measurements of extracellular polyamine levels (Figure 4), PUT is converted to GABA, which is released into the extracellular space (denoted by larger font sizes). SPD synthase inhibition also reduces SPD concentration (denoted by smaller font size). Levetiracetam, on the other hand, can increase the surface expression of GAT-2/3 transporters through which astrocytic GABA is released from astrocytes, generating larger tonic currents.

Although several studies have reported correlation between brain SPD/SPM levels and epileptic activity (Laschet et al., 1992, 1999; Leonetti et al., 2020), the molecular mechanisms by which polyamine metabolism contributes to the generation and/or maintenance of seizures are not detailed as yet. In this study, we explored whether various interventions modifying the astrocytic polyamine concentrations can be effective against epileptiform discharges in the low-[Mg^2+^] *in vitro* model of TLE and in the *in vivo* WAG/Rij rat model of absence epilepsy. We showed that inhibiting the conversion of PUT to SPD drastically suppresses epileptic seizures, most likely by stimulating astrocytic GABA synthesis from PUT. To interpret these results, we propose a scheme that elucidates how astrocytic polyamine metabolism and the coupled GABA release may shape epileptogenesis (Figure 6).

The polyamines SPD and SPM are synthesized from low molecular weight polyamines, agmatine, and PUT (Krauss et al., 2006; Peters et al., 2013; Piletz et al., 2013; Pegg, 2014). Intracellularly, the brain contains high amounts of SPD/SPM while PUT is present at much lower concentrations, approximately 2% of total polyamine content (Shaw and Pateman, 1973; Shaw, 1979), which is further declining with age (Shaskan, 1977). Majority of SPD is found in zones enriched with glial cells such as white matter and brainstem (Shaw and Pateman, 1973). SPD is arranged at a central position in the polyamine metabolism. It is synthesized from PUT by SPD synthase and can be converted to SPM by SPM synthase. Since PUT can be converted to GABA, which increases tonic inhibition (Héja et al., 2009, 2012; Unichenko et al., 2013; Wójtowicz et al., 2013), amplification of polyamine metabolism upstream to SPD is inhibitory in nature. In contrast, downstream metabolism of SPD produces SPM, which directly increases excitation (Figure 1) and also enhances synchronization by opening gap junctions (Benedikt et al., 2012). The upstream and downstream metabolic pathways, therefore, are expected to play anticonvulsive and pro-convulsive roles, respectively (Figure 6). It is to note, however, that opening of astrocytic gap junctions and subsequent enhancement of synchronization, surprisingly attenuates seizures in absence epilepsy (Vincze et al., 2019). Therefore, in WAG/Rij rats, even the downstream SPD metabolism can induce enhanced epileptiform activity.

By blocking SPD synthesis with the SPD synthase inhibitor 4-MCHA, we observed complete elimination of seizures in WAG/Rij rats (Figure 3A). This observation can be explained by either the increased GABA production or the antiepileptic effect of decreased gap junction opening due to reduced SPM concentration (Figure 6). To differentiate between the two explanations, we specifically blocked SPM synthase activity with APCHA. Since this approach did not lead to SWD reduction (it even temporarily increased the frequency of seizures), we suggest that 4-MCHA exerted its effect mainly by increasing astrocytic PUT level and corresponding enhancement of tonic inhibition. This hypothesis is also supported by the identified mechanism of the antiepileptic action of the FDA-approved drug levetiracetam. Levetiracetam is effective against absence epilepsy, but the mechanism by which it attenuates seizures is not well-understood (Surges et al., 2008). Importantly, levetiracetam has been shown to increase the surface expression of GAT-2/3 transporters, through which GABA is released from astrocytes (Doi et al., 2005; Ueda et al., 2007). In this study, we show that the application of the specific GAT-2/3 transporter blocker SNAP-5114 reversed the antiepileptic effect of levetiracetam (Figure 5), suggesting that the GAT-2/3 expression enhancement is the major route through which levetiracetam exerts its antiepileptic effect.

It is noteworthy to mention that the source of astrocytic PUT is likely different in our low-[Mg^2+^] and WAG/Rij seizure models due to the different age of the animals used. We have previously showed (Héja et al., 2012) that ornithine to PUT conversion is increased in the low-[Mg^2+^] model in juvenile (P11-15) rats. In contrast, inhibition of this route by DFMO did not significantly alter SWD appearance in adult (>8 month) WAG/Rij rats. This observation most likely corresponds to the declined metabolic production of PUT in older animals (Ramos-Molina et al., 2019) and critical dependence of astrocytic polyamines on transport, observed in adults.

It has been demonstrated that absence epileptic activity can be provoked by increasing the inhibitory tone by GABAergic agonists or GABA uptake blockers (Peeters et al., 1989; Coenen et al., 1995). This mechanism likely occurs *via* membrane hyperpolarization in thalamic relay neurons, which process is necessary to evoke SWDs through burst firing mode (Murray-Sherman, 2001; Cope et al., 2009). However, according to the cortical focus theory of absence epilepsy genesis (Meeren et al., 2002), spontaneously occurring SWDs in WAG/Rij rats are triggered by hyperexcitable neurons in the somatosensory cortex. Therefore, although increased tonic inhibition in the thalamus may be pro-epileptic, enhanced GABA release from astrocytes can hyperpolarize the neuronal membrane and decrease excessive hyperexcitability in the somatosensory cortex and consequently can eliminate seizure generation even before the thalamic network became activated. Indeed, increase in astrocytic GABA level (Figure 4) combined with GAT-2/3 transporter overexpression (Figure 5) can enhance GABA release and decrease SWD number (Figures 3, 5) in WAG/Rij rats. The total elimination of seizures by inhibition of SPD synthase activity (Figure 3A) also supports this hypothesis.

In summary, we identified the inhibition of PUT to SPD conversion and the enhancement of the corresponding GABA synthesis from PUT (Figure 6) as an effective target mechanism to combat both convulsive (TLE) and non-convulsive (absence epilepsy) seizures.

Theoretically, modulation of glial PUT-SPD-SPM metabolism may be a promising therapeutic tool in the treatment of not only epilepsy but also other CNS diseases. In this study, we showed (Figure 1) that metabolic inhibition of astrocytes by the specific gliotoxin FA (Keyser and Pellmar, 1994, 1997; Fonnum et al., 1997) resulted in massive loss of SPM in astrocytes, indicating astrocytic SPM release. This observation is consistent with a switch of PPI to PPF by SPM in neuronal network (Figures 1B,C), resulting in disinhibitory (proconvulsant) SPM effect. Noteworthily, animals over-expressing SPM oxidase (SMOX) (Cervelli et al., 2013) developed epileptic seizures and oxidative stress (Leonetti et al., 2020). Reportedly, over-expression of SMOX caused robustly increased activity most probably associated with the release of Glu from Bergman gliosomes (Cervetto et al., 2015, 2016) and release of SPM (Cervetto et al., 2021). We may raise the possibility that clinical seizures and Snyder-Robinson syndrome, which is the only known genetic disorder is associated with the polyamine metabolic pathway. Explicitly, the syndrome features SPM synthase deficiency, thus excessive SPD catabolism may generate toxic metabolites, lysosomal defects and oxidative stress (Li et al., 2017). Intriguingly, depressive/suicidal completers also show disturbance in polyamine machinery, particularly in the level of expression and mutations of SSAT enzyme degrading polyamines (Sequeira et al., 2006). Using sufficient statistics (181 male suicide completers and 80 male controls), the authors found mutation in SSAT342C allele among suicide cases, suggesting that this allele may increase predisposition to suicide (Sequeira et al., 2006). SSAT produces acetylated polyamines, and the increase of acetylated polyamines with decline in blood plasm SPM content and SPM/SPD ratio were found in aging and specifically in patients with Parkinson’s disease (Saiki et al., 2019). On the other hand, supplements with SPD delay brain aging and improve cognitive functions (Sigrist et al., 2014; Wirth et al., 2018, 2019, 2021; Maglione et al., 2019; Xu et al., 2020). Dietary SPD intake increases brain volume specifically in brainstem, hippocampus, and cortex of old human (Schwarz et al., 2020), and in mice, dietary SPD passes the blood-brain barrier and increases hippocampal eIF5A hypusination, mitochondrial function, improves spatial learning, and increases hippocampal respiratory competence. Since glial cells are major holders of polyamines and are participants of neuronal-glial network, the role of glial polyamines can be reconsidered in stress and aging (Skatchkov et al., 2014, 2016; Kirichenko et al., 2021).

## Data Availability Statement

The raw data supporting the conclusions of this article will be made available by the authors, without undue reservation.

## Ethics Statement

The animal study was reviewed and approved by the Government Office for Pest County, Hungarian National Scientific Ethical Committee on Animal Experimentation, Universidad Central del Caribe Institutional Animal Care and Use Committee.

## Author Contributions

JK, LH, RV, and SS: conceptualization. LH, ZK, ZS, RV, SS, PS, and JK: methodology and formal analysis. ZK, ZS, RV, SS, KN, and PS: investigation. JK and LH: writing and original draft preparation. LH: visualization. JK, SS, and LH: supervision, project administration, and funding acquisition. ZK, SS, RV, ZS, KN, PS, JK, and LH: data curation and validation and writing, review, and editing. All authors have read and agreed to the published version of the manuscript.

## Conflict of Interest

The authors declare that the research was conducted in the absence of any commercial or financial relationships that could be construed as a potential conflict of interest.

## Publisher’s Note

All claims expressed in this article are solely those of the authors and do not necessarily represent those of their affiliated organizations, or those of the publisher, the editors and the reviewers. Any product that may be evaluated in this article, or claim that may be made by its manufacturer, is not guaranteed or endorsed by the publisher.
